# The Tactical Employment of the Medical Service in War Operations

**Published:** 1910-08-27

**Authors:** 


### The Royal Army Medical Corps Section.

### THE TACTICAL EMPLOYMENT OF THE MEDICAL SERVICE IN WAR OPERATIONS.

In The Hospital for April 16 we dwelt on the importance and the necessity of military training for medical officers, and detailed the steps by which the authorities think it should be carried out. Of the means suggested we pointed out that the two known as War Games and Staff Tours are undoubtedly the best for educational purposes, and in fact they are very closely allied, the Staff tour (or ride) being a practical application of what is worked out theoretically at a war game.

They are both primarily intended for the instruction of medical officers either in their own duties or in the study of strategical or tactical problems. It is in connection with the latter that these exercises are so valuable, and it is laid down that, should circumstances permit, every officer of the R.A.M.C. of the rank of captain and upwards must attend at least one special Staff tour annually, and that field officers will, in addition, attend at least one Staff tour each year. What is regarded as so necessary for medical officers of the R.A.M.C. is also considered important for those of the Territorial Medical Service, and the authorities are giving facilities to these latter for taking part in this form of instruction. Applications for such Staff tours should be made through the General Officer Commanding the Division, who obtains the necessary permission and money required for carrying them out. The tours extend over a period of two to four days, and officers attending them are paid their travelling expenses, with 15s. a day detention allowance for all ranks to meet hotel and other charges.

Staff Tours.

The method of working such a Staff tour is to assemble the medical officers at some definite centre, where they arrive in the evening and have a preliminary lecture explanatory of the area of operations and their objective. Next day the officers go over the ground and have the position of the contending forces shown to them. Notes of the information so supplied are made, and then an appreciation is furnished on the whole situation by each individual officer attending, who indicates what his medical arrangements would be in the circumstances, and his reasons for them. In the case of Staff tours arranged solely for the instruction of medical officers there is placed in charge a selected medical officer of the R.A.M.C., whose duty it is to criticise the work of the medical officers taking part in the tour, and to furnish full explanations as to what should be the medical arrangements suitable for the situation under consideration. This plan of practising medical officers during peace time in adapting their arrangements to military requirements based on definite situations is a most excellent method of instruction, as it furnishes opportunities for a detailed study of medical problems in the field, and gives practice in the actual disposition and movement of medical units to meet the varying conditions of warfare. In connection with this very important subject of training medical officers in the duty of framing their medical assistance on definite tactical situations we are glad to see the issue by the War Office of a translation by Lieut.-Colonel W. G. MacPherson, C.M.G., R.A.M.C., of Ritter von Hoen's volume on "The Strategical and Tactical Employment of the Medical Service as carried out in an Army Corps" (*Der operative und taktische Sanitätsdienst im Rahmen des Korps*). Colonel von Hoen is on the Staff of the Austro-Hungarian Army, and is Instructor in the Imperial and Royal Army Medical College, Vienna. Four years ago he published an "Introduction to the Study of Problems connected with the Tactical Employment of the Medical Service" (*Vorschule zur Lösung Sanitätstaktischer Aufgaben*), which contained in three parts thirty-eight lectures on army organisation, march formations, map-reading, Staff tours, war games, and other exercises. The success attending this publication has induced him to supplement his "Introduction" by a volume which is more advanced, and embraces a wider and deeper study of what for brevity's sake is usually spoken of as "Medical Tactics"; that is to say, the proper use of medical units when face to face with the enemy, and when the medical officer in charge is called upon to make arrangements that are best suited to meet the conditions that are present or may develop in the course of or as the result of actual fighting. A little reflection will show that this may be no easy problem to decide. When in battle array the regiments of a division or a brigade may be disposed in very different ways, and the area over which they are acting may be very wide or very limited. They may have duties assigned to them that may necessitate their rapid advance, or retirement, or their retention of a position at all costs. Seeing that the assistance in the field comprises not only "first-aid" assistance by the Field Ambulance units, but the preparation for the evacuation of the wounded subsequent to the engagement, it is not difficult to see that the distribution of the medical resources at the point where the greatest number of casualties may occur, and the provision of adequate transport for their removal, will call for careful consideration and a practical knowledge of the work that cannot come intuitively. This ability to bring the various medical units to the spot where the chief contact with the enemy takes place, and to employ them so that they will most quickly carry out the necessary aid to the wounded, lies at the root of efficient medical service in war.

Colonel von Hoen's latest volume is in two parts, of which the first one deals with the theory of the strategical and tactical employment of the medical service as it affects the principal medical officer of an army corps or of a division, while separate chapters are also devoted to the tactical work of the officer in command of a divisional medical unit, and to medical service with regimental units. The second part contains a series of problems embodying certain general and special situations which have to be met. These are worked out in detail, and by the help of excellent maps supplied the different points are made clear and instructive. In fact the whole ground, from the period of strategical concentration of the fighting force to the final issue of a decisive battle, is covered, and the varying conditions that may have to be met are distinctly provided for. No doubt the army medical organisation of the Austro-Hungarian army differs in many essential details from that of the British, but underlying them both is the same working basis of quick evacuation of the sick and wounded. The efficiency with which this is arranged for and carried out greatly affects the mobility and moral of the army, because, as the introduction to von Hoen's book says, "this is best for the wounded themselves, as well as for the fighting force." We draw attention of medical officers to this publication because we fully endorse what the translator puts forward in justification of his work when he says that "it will help to instruct officers of the Royal Army Medical Corps and others, because it will encourage thought on a subject which, in consequence of training by means of Staff tours, is attaining greater importance every day, and because it presents a wider view of the duties and responsibilities which must fall on all those who have to decide upon measures for the care of sick and wounded than can be obtained by a training which is confined to first-aid and ambulance drill only."

